# High-density P300 enhancers control cell state transitions

**DOI:** 10.1186/s12864-015-1905-6

**Published:** 2015-11-06

**Authors:** Steven Witte, Allan Bradley, Anton J. Enright, Stefan A. Muljo

**Affiliations:** Integrative Immunobiology Unit, Laboratory of Immunology, National Institute of Allergy and Infectious Diseases, National Institutes of Health, Bethesda, MD USA; Wellcome Trust Sanger Institute, Wellcome Trust Genome Campus, Hinxton, Cambridge UK; EMBL - European Bioinformatics Institute, Wellcome Trust Genome Campus, Hinxton, Cambridge UK

**Keywords:** Transcriptional regulation, Lysine acetylation, Epigenetics, Long non-coding RNA, Systems biology, Inflammation, Cell differentiation, Super-enhancers

## Abstract

**Background:**

Transcriptional enhancers are frequently bound by a set of transcription factors that collaborate to activate lineage-specific gene expression. Recently, it was appreciated that a subset of enhancers comprise extended clusters dubbed stretch- or super-enhancers (SEs). These SEs are located near key cell identity genes, and enriched for non-coding genetic variations associated with disease. Previously, SEs have been defined as having the highest density of Med1, Brd4 or H3K27ac by ChIP-seq. The histone acetyltransferase P300 has been used as a marker of enhancers, but little is known about its binding to SEs.

**Results:**

We establish that P300 marks a similar SE repertoire in embryonic stem cells as previously reported using Med1 and H3K27ac. We also exemplify a role for SEs in mouse T helper cell fate decision. Similarly, upon activation of macrophages by bacterial endotoxin, we found that many SE-associated genes encode inflammatory proteins that are strongly up-regulated. These SEs arise from small, low-density enhancers in unstimulated macrophages. We also identified expression quantitative trait loci (eQTL) in human monocytes that lie within such SEs. In macrophages and Th17 cells, inflammatory SEs can be perturbed either genetically or pharmacologically thus revealing new avenues to target inflammation.

**Conclusions:**

Our findings support the notion that P300-marked SEs can help identify key nodes of transcriptional control during cell fate decisions. The SE landscape changes drastically during cell differentiation and cell activation. As these processes are crucial in immune responses, SEs may be useful in revealing novel targets for treating inflammatory diseases.

**Electronic supplementary material:**

The online version of this article (doi:10.1186/s12864-015-1905-6) contains supplementary material, which is available to authorized users.

## Background

Recently, clusters of enhancers termed stretch- or super-enhancers (SEs) were identified as large *cis*-acting elements in the genome that uniquely regulate cell lineage-specific gene transcription and define cell identity [1, 2]. SEs are characterized by potent transcriptional enhancer activity that correlates with high occupancy by transcription factors (TFs) and co-activators, and pronounced sensitivity to perturbation [3]. Additionally, non-coding disease-associated genetic variation is enriched in SEs [2, 4]. To identify SEs, the binding density of Med1, a subunit of the transcriptional Mediator protein complex, was originally used [1, 3]. Similarly, Brd4, a member of the bromodomain and extraterminal (BET) family of transcriptional coactivators that interact with Mediator, can also be used to identify SEs [3].

Since SEs are central to cell identity, they may help find factors for direct reprogramming of cells, or identify drug targets to treat diseases. We explore this latter notion by analyzing P300 density at enhancer sites in cell transition states. Pioneering studies have demonstrated that chromatin immuno-precipitation followed by deep sequencing (ChIP-seq) can reliably measure binding of P300, a histone acetyltransferase and transcription activator, across the genome to predictively map transcriptional enhancer elements active *in vivo* [5–7].

Although SEs have been shown to regulate cell identity genes in resting steady state, the role of SEs in regulating transition states such as cell differentiation and activation is not well appreciated. Several epiblast stem cell SEs have been reported to arise from ‘seed’ enhancers in embryonic stem cells [8, 9], however this observation has not been analyzed systematically in other cell types. Since these transition states are frequently targeted in therapies for inflammatory disease and cancer, we characterized the dynamic regulation of enhanceosome assembly using the well known transcriptional co-activator protein P300. First, we establish P300 as a reliable mark of SEs, then show that high-density P300 SEs are induced in cell transition states and are located near cell identity and disease susceptibility genes.

We also find that non-coding transcription is often associated with SEs. Long non-coding RNAs (lncRNAs), which associate with chromatin modifying complexes and play roles in gene expression regulation either in *cis* or *trans* [10, 11], are much more likely to be located in proximity to SEs than conventional enhancers (CEs). LncRNAs are RNA transcripts greater than 200 base pairs in length, and are often spliced and polyadenylated. A category of non-coding RNAs arising from enhancer regions, known as enhancer RNAs (eRNAs), participate in enhancer activity and regulate neighboring genes [12]. These eRNA transcripts often lack polyadenylation, as well as the typical H3K4me3 promoter signature present in the loci for other classes of lncRNA genes [11, 13, 14]. Here, we show that some eRNAs are coincident with some SE regions.

Finally, we found that the TLR4 stimulated inflammatory response led to drastic remodeling of SEs in macrophages. Many SE-associated genes are highly induced, suggesting that inflammation can be targeted by blocking SEs. Small molecule BET inhibitors effectively disable SEs in tumor cell lines and stop cell growth [3], and we show that chemical inhibitors that abrogate SE function also block expression of inflammatory genes, as well as affect cell fate decisions in macrophages and T helper cells.

## Results

### Characterization of P300-marked super-enhancers

**Fig. 1:**
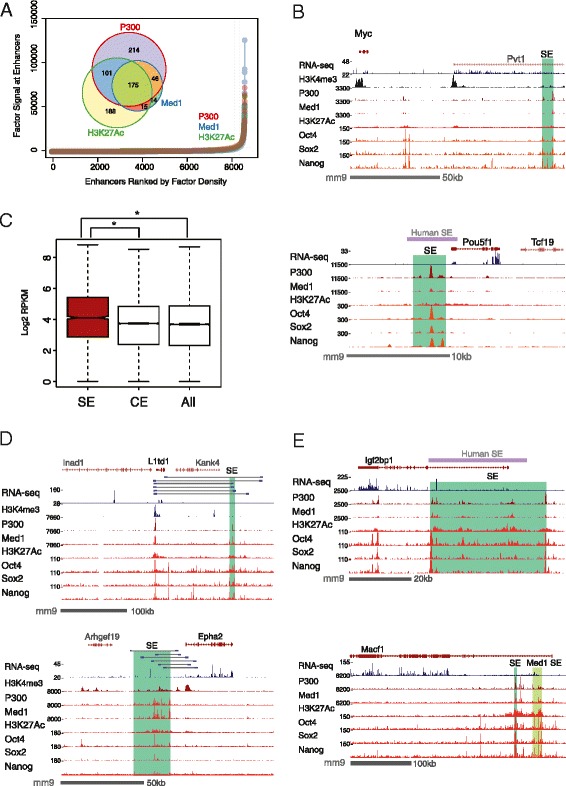
Identification of P300 dense super-enhancers. **a** Ranked distribution plot of P300, Med1, or H3K27ac binding density identifies a small subset of SEs (above the inflection point) in mouse ESCs. Venn diagram (inset) shows the overlap between P300, Med1, and H3K27ac marked SEs. ChIP-seq data are from [71, 72]. **b** Genome browser screenshot depicting mESC RNA-seq and ChIP-seq data as labeled. (Top panel) The *Myc* gene is adjacent to *Pvt1*, a lncRNA overlapping with a SE identified by P300, Med1 and H3K27ac. (Bottom panel) Oct4 is encoded by the *Pou5f1* locus which harbors a SE marked by P300, Med1, and H3K27ac. In addition, P300 co-localizes with the core pluripotency factors Oct4, Sox2, and Nanog in both cases. **c** Expression of genes near P300 SEs and CEs. Genes that are located within 100 kb of a SE are expressed at higher levels than those near a CE, and all expressed genes. **d** (Top) ChIA-PET reveals that a P300 SE loops ~100 kb to physically associate with the promoter (marked by H3K4me3) of the ESC-specific *L1td1* gene. (Bottom) Chromatin interaction between a SE and a gene highly expressed in mESCs, *Epha2*. ChIA-PET data are from [20] and depicted as grey lines with blue ends. **e** In mESCs, 315 additional SEs were identified from P300 density than from Med1 density. *Igf2bp1* is shown as an example of a P300-specific SE (upper panel). In contrast, there are only 29 SEs identified by Med1 in mESCs but not by P300. For example, a Med1-specific SE near the gene *Macf1* is shown (bottom panel). There are two SEs in close vicinity to *Macf1*; both were identified as SEs by Med1, while only one qualified as a SE by P300

P300, also known as E1A-binding protein, 300 kD (EP300), has been previously used to identify enhancers [5–7], and it was recently suggested that P300 may also mark SEs [4, 15]. To determine whether P300 can be used to identify SEs, we analyzed P300, Med1, and H3K27ac ChIP-seq data in mouse embryonic stem cells (mESCs). We found that P300 is especially dense at previously reported SE sites, including the important pluripotency genes *Pou5f1* and *Sox2* (Fig. 1b, Additional file 1: Figure S1d). When enhancers were ranked by density, P300 had a similar distribution plot to Med1 and H3K27ac, recovering 88 % (221/250) of Med1 SEs (Fig. 1a). There is a high correlation of P300 and Med1 at these shared sites (Additional file 1: Figure S1A). The non-coding *Pvt1* gene neighboring *Myc* that harbors a SE was recently shown to contribute to high expression of this locus in cancer cells [16]. A P300-marked SE overlaps this locus, and may contribute to its expression in mESCs (Fig. 1b, upper panel). Genes near SEs are highly expressed in mESCs relative to genes associated with conventional enhancers (CEs) (*p*-value ≤ 2e-11, Students *t*-test) and all expressed genes on average (*p*-value ≤ 3e-13, Students *t*-test) (Fig. 1c). We also observed that SEs are closer to transcription start sites than CEs are, on average, and it is possible that this property is related to the increased gene expression associated with SEs (Additional file 2: Table S1B). Furthermore, SEs are large on average, as expected (Additional file 1: Figure S1C). In the examples that we show, the P300 SEs are co-bound by Oct4, Nanog, and Sox2 (Fig. 1b, d, e and Additional file 1: Figure S1D), which has been reported to be a feature of SEs in general in mESCs [17].

P300 SEs participate in long-range chromatin interactions by looping to promoters (Additional file 1: Figure S1E), a known mechanism of action for transcriptional enhancers and SEs [18]. For example, we show such an interaction between the promoter of a stem cell self-renewal gene *L1td1* [19] and a SE 110 kb 3' (Fig. 1d, upper panel), detected from RNA-polymerase II ChIA-PET data [20]. The gene *Kank4* is located in between the SE and *L1td1* but appears to be completely bypassed and accordingly is not expressed in mESCs. As another example, we detect chromatin interaction between the promoter of *Epha2* and a SE 15 kb 5' (Fig. 1d, lower panel). Finally, we found that P300 was able to detect one putative SE that was not detected by Med1 in mESCs (Fig. 1e) [21]. It is possible that other SEs may not have been detected by Med1, however this remains to be determined. This suggests that P300 may assist in identifying additional SEs that are important biological targets not found in previous analysis using Med1 alone. Binding of P300, Med1, and H3K27ac is present in this SE, suggesting that SEs are marked by all three factors; however, they do not necessarily have enough factor density to make the SE cutoff for all three factors. A closely related paralog to P300, CBP, has also been used to mark SEs [4, 22], and we show that it can identify a highly similar SE repertoire as P300, suggesting that these two factors have similar associations with SEs (Additional file 1: Figure S1F).

Since P300 ChIP-seq appears to be a robust method for systematic identification of SEs, we analyzed all available P300 ChIP-seq data sets from multiple cells and tissues, in both human and mouse, and integrated the results with previously reported SEs, increasing the total number of SE datasets to >200 (Additional file 2: Table S1). Datasets for the P300 paralog CBP are included as well [4, 22]. These catalogs can be explored via our website (http://www.serbase.org). Next, we demonstrate the power of this resource using T helper cells and macrophages as systems undergoing cell state transitions.

### Role of SEs in T helper cell gene expression programs

Th1, Th2 and Th17 cells differentiate from a common progenitor, naïve CD4^+^ T helper cells. They are important in the adaptive immune response and many immune-related diseases. Lineage specification of T helper cells first requires cell activation, provided by stimulation of the T cell antigen receptor and co-stimulatory molecules such as CD28, as well as cytokine signaling. T helper cells serve as a well-characterized model system for understanding cell differentiation [23].

**Fig. 2:**
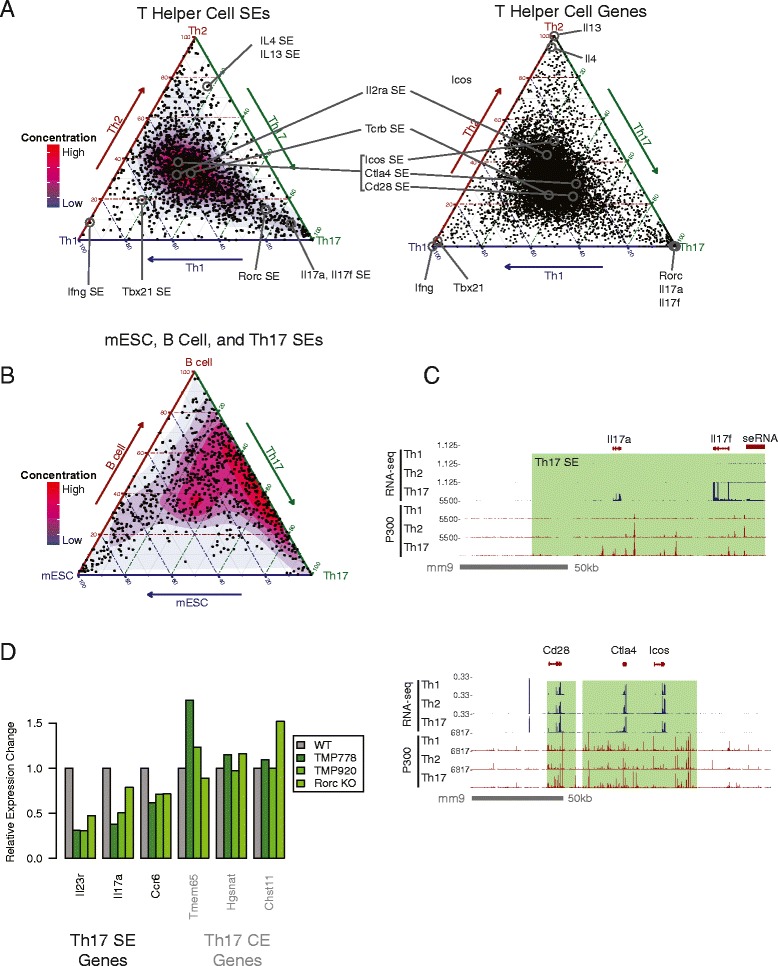
Super-enhancers in T helper cells. **a** Ternary plot of P300 density at SEs in Th1, Th2, and Th17 cells (left) or gene expression in the same cells (right). Each dot is a SE or gene. T helper cell SEs are largely shared between all three subsets. Axes indicate relative factor density between Th1, Th2, and Th17 cells. Color scale indicates concentration of SEs (note: area of graph occupied by color scale for gene expression is much smaller than area of color scale for P300 density, and is not visible because it is covered by overlapping points). ChIP-seq data are from [24, 25]. RNA-seq data are from [26]. **b** Ternary plot of P300 density at SEs in mESC, stimulated B cells, and Th17 cells. Color scale indicates concentration of SEs. Some SEs are shared between B cells and Th17 cells, but are distinct from mESCs, a distantly related cell lineage. B cell ChIP-seq data are from [74]. **c** (Top) *Il17a* and *Il17f* are regulated by a Th17-specific SE spanning both genes. Also, a non-coding RNA is transcribed 3' of *Il17f*. (Bottom) *Cd28*, *Ctla4* and *Icos* are regulated by two SEs active in Th1, Th2, and Th17 cells. Similar levels of expression and P300 density are seen in all three cell types. **d** Genes from both the Th17 SE module and the Th17 gene expression module which have lower expression when treated with the RORγt inhibiting drugs, TMP778 or TMP920. They are also expressed lower in RORγt-deficient Th17 cells. CE-genes from Th17 cells are generally unaffected (SE examples were chosen because of known disease relevance, CE examples shown were chosen arbitrarily). Data are from [27]

To determine how SEs regulate gene expression programs during T helper cell differentiation, we integrated ChIP-seq and RNA-seq data from various studies [17, 24–27] (Additional file 3: Table S2). We found that 44 % of SEs were shared by all three T helper cell subsets and 75 % by two or more cell types (Fig. 2a and Additional file 4: Figure S2A). When we compared more distantly related cells, less SEs were shared: only 10 % of SEs were shared between Th17 cells and mESCs, and 27 % were shared between Th17 cells and activated B cells (Fig. 2b).

To see if SEs are associated with cell-specific gene expression, we divided T helper cell SEs and expressed genes into seven modules based on cell specificity (see Additional file 4: Figure S2B for module definitions). When SEs were compared to expressed genes in a corresponding module (for example, Th1 specific SEs compared to Th1 specific genes), strong enrichment was seen: Th1 genes were closer to Th1-unique SEs (*p*-value ≤ 2e-8, Students *t*-test). In Th2 cells, Th2 genes were closer to Th2-unique SEs (*p*-value ≤ 7e-32, Students *t*-test), and Th17 genes were closer to Th17-unique SEs (*p*-value ≤ 1e-13, Students *t*-test).

As validation, known signature cytokines and transcription factors were found in this enrichment analysis. For example, *Tbx21* and *Ifng*, two signature Th1 genes, are found near SEs that are inactive in Th2 and Th17 cells, while *Rorc* and *Il17a*, Th17 signature genes, are near Th17-unique SEs (Fig. 2a, c). *Cd28, Icos*, and *Ctla4* share expression in Th1, Th2, and Th17 cells, and P300 SEs are seen in all three cell types (Fig. 2c bottom). Overall, 4,596 of all 13,194 protein-coding genes expressed in T helper cells were located within 100 kb of a T helper cell SE (accounting for 1742/1972 T helper cell SEs). A list of SE-associated genes in T helper cells is provided in Additional file 5: Table S3.

The transcription factors STAT3, RORγt, IRF4, and BATF are required to drive the Th17 gene expression program [25]. To determine whether these transcription factors control cell fate through SEs, we analyzed RNA-seq expression data from Th17 cells treated with the RORγt inhibitors TMP778 and TMP920 [27], and Th17 cells missing key transcription factors [25]. SE-associated genes from the Th17 modules, such as the autoimmune susceptibility genes *Il23r* and *Il17a*, were strongly down-regulated as a result of these perturbations, and we hypothesize that RORγt, STAT3, and possibly IRF4 and BATF may be important for mediating Th17 SE activity (Fig. 2d and Additional file 4: Figure S2D-E). Further analysis of the constituent regions of SEs in Th17 cells indicates that SEs are highly enriched for sites co-bound by STAT3, BATF, IRF4, c-MAF and RORγt (*p*-value < 2.2e-16, Pearson’s Chi-squared test) (Additional file 4: Figure S2D). STAT3 is capable of recruiting P300 [28] and may play a direct role in assembly of super-enhanceosomes. In Th17 cells missing STAT3, for example, there is a greater decrease in expression of genes associated with SEs than CEs (Additional file 1: Figure S1E, *p*-value ≤ 0.02, Students *t*-test).

### Transcription of non-coding RNAs in relation to SEs

**Fig. 3:**
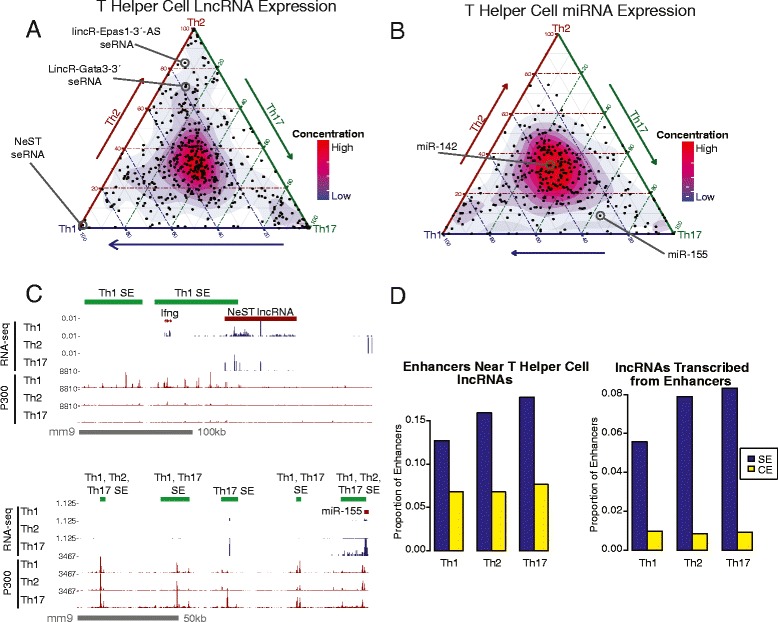
Non-coding RNAs are frequently near or overlapping SEs. **a** Ternary plot of lncRNA expression in Th1, Th2, and Th17 cells. Color scale indicates concentration of lncRNAs. Notable examples of lncRNAs that are lineage specific and located near SEs are shown. RNA-seq data are from [26]. **b** Ternary plot of miRNA expression in Th1, Th2, and Th17 cells. Color scale indicates concentration of miRNAs. MiR-155 and miR-142 are labeled as examples of miRNAs located near SEs. Small RNA-seq data are from [29]. **c** LncRNAs expressed from SEs are cell-type specific. (Upper panel) NeST, a lncRNA (or eRNA) that is expressed from the SE at the *Ifng* locus. NeST, *Ifng*, and the SE are active in Th1 cells, but not in Th2 or Th17 cells. (Bottom pane) miR-155 is expressed in Th17 cells and is located near multiple SEs that are active in T helper cells. **d** A SE is more likely than a CE to be associated with a lncRNA. Proportion of SEs or CEs that are located within 100 kb of a lncRNA expressed in Th1, Th2, or Th17 cells (left). Proportion of lncRNAs (or eRNAs) that are directly transcribed from SEs or CEs, in Th1, Th2, and Th17 cells (right) (p-value < 2.2e-16 for all three comparisons)

Since non-coding RNAs are emerging as integral components of genetic regulatory networks, we explored their association with SEs in T helper cells. We looked at miRNAs and lncRNAs, two classes of non-coding RNA that are known to be cell specific and to be involved in gene regulation. We divided T cell lncRNAs and miRNAs into subset-specific modules (see Additional file 4: Figure S2B for module definitions) and looked at enrichment for SEs [26, 29] (Fig. 3a, b; list of lncRNAs and miRNAs in SE modules in Additional file 6: Tables S4 and Additional file 7: Table S5 respectively). Overall, enrichment was higher than for protein-coding genes, as 63 % of lncRNAs vs 47 % of protein-coding genes were enriched for SEs (*p*-value < 0.026, Pearson’s Chi-squared test).

We report that three previously described lineage-specific T cell lncRNAs are in fact transcribed from lineage-specific SEs. For example, NeST is a lncRNA important for protection from *Salmonella* [30] that is transcribed from a SE 3' of *Ifng* in Th1 cells (Fig. 3c top). Interestingly, although the NeST SE is moderately enriched for P300 in Th2 and Th17 cells, it appears to only be active in Th1 cells. Additionally, there were two Th2-specific lncRNAs that were associated with Th2 SEs: lincR-Epas1-3'-AS and lincR-Gata3-3' (Fig. 3a). These lncRNAs are regulated by STAT6 in Th2 cells [26].

Next we focused on lncRNAs that are directly transcribed from SEs, as SEs were much more likely to have lncRNA transcription than CEs (8.3 % of SEs vs. 0.9 % of CEs in Th17 cells, *p*-value < 2.2e-16, Pearson’s Chi-squared test) (Fig. 3d). For example, a SE overlapping *Ccr7* has at least three non-coding transcripts arising from it (Additional file 8: Figure S3A). Notably, two regions of the *Ccr7* SEs are conserved between mouse and human (Fig. 3e). The expression of *Ccr7* and its lncRNAs are reduced in the absence of STAT3 and BATF, TFs that bind throughout this locus, suggesting that they are directly regulating transcription of this locus. CCR7 is an important chemokine receptor for T cell lymph-node homing, and has a role in T cell mediated pathogenesis of inflammatory disease [31, 32]. It is a possibility that many of these transcribed regions within SEs represent eRNAs, however additional datasets such as GRO-seq are necessary in order to validate this.

A microRNA, miR-142, highly expressed in all T helper cell subsets, is associated with a shared SE (Fig. 3b). In contrast, miR-155, appears to be regulated by a conditional Th17-specific SE (Fig. 3b, c), and as such, miR-155 has a pro-inflammatory role in Th17 cells [33]. The *Mir155* locus contains moderate P300 binding in all three T helper cell lineages, despite the fact that expression of miR-155 is highest in Th17 cells. However, one peak located 68 kb 5' of miR-155 is highly enriched in Th17 cells relative to Th1 and Th2 cells, and may be responsible for the cell lineage specificity of this gene.

### Pharmacologic perturbation and genetic variation of SEs blocks inflammatory response

**Fig. 4:**
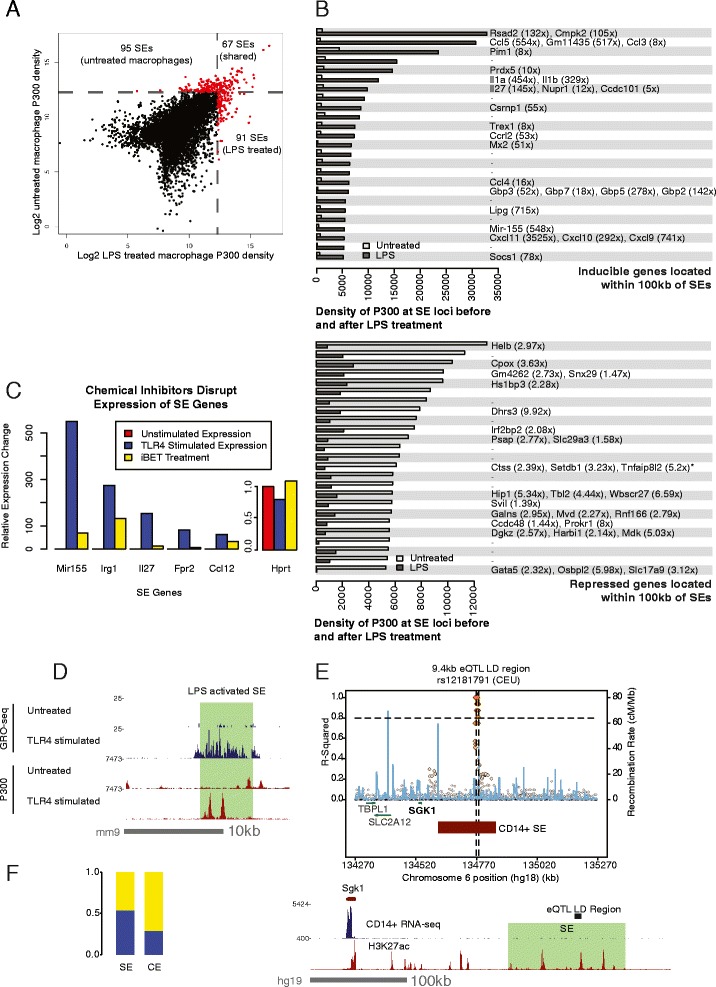
Super-enhancers in unstimulated and activated macrophages. **a** A scatterplot of P300 density at enhancer sites in resting versus stimulated macrophages. **b** The density of P300 at many enhancers and SEs is dynamically changed upon stimulation of macrophages. ChIP-seq data are from [36]. Analysis of top 25 most highly induced SEs upon macrophage activation (top) or repressed SEs (bottom). Density of P300 in activated macrophages is shown compared to resting macrophages. For those SEs located within 100 kb of a macrophage inducible gene, the name of the gene(s) is shown along with the fold change in expression (Top: increase in fold change, bottom: decrease in fold change). The absence of a gene name denotes the absence of an annotated protein-coding gene within 100 kb of the SE. RNA-seq data are from [34]. *continued from figure due to lack of space: Fam63a (4.38x), Golph3l (2.19x), Lysmd1 (4.92x), Prune (5.89x), Scnm1 (2.13x), Vps45 (2.46x), Tmod4 (4.26x). **c** The expression of many genes from (B) is blocked when TLR4-stimulated macrophages are treated with iBET. GRO-seq data are from [35]. **d** A SE near *Pim1* is induced following TLR4 stimulation. GRO-seq reveals that this SE is also transcribed when active. **e** (Top) Shown is a local association plot for a cis-eQTL that regulates the *SGK1* gene. The most significantly associated SNP from the linkage disequilibrium (LD) region is rs1281791. Dotted line designates the R-squared value of 0.8 used as a cutoff for determining linked SNPs. Blue line indicates genomic recombination rate (y-axis on right). The entire LD region is also located within a SE active in human CD14^+^ monocytes. eQTL data are from naïve CD14^+^ cells [49]. (Bottom) Expression of SGK1 in human CD14^+^ monocytes, along with H3K27ac density (data from GSE18927). **f** Enrichment of human monocyte eQTLs in SEs vs. CEs. 550/1019 monocyte SEs overlapped a naïve monocyte eQTL LD region (54 %), compared to only 5471/19028 CEs (29 %). eQTL data are from [49]

We analyzed SEs in unstimulated and stimulated mouse macrophages and explored their relation to inflammatory gene expression (data from [34–36]). Macrophages activated by TLR4 signaling had a strikingly different SE profile than unstimulated macrophages. In all, only 26 % (67/253) of SEs were shared between the two macrophage states (Fig. 4a). Genes that increase expression following TLR4 stimulation were closer to an induced SE than pre-existing SEs (*p*-value ≤ 0.005, Students *t*-test) or no SE (*p*-value ≤ 0.01, Students *t*-test) (Additional file 9: Figure S4A). The top 25 most highly induced or repressed SEs in activated macrophages were examined further (Fig. 4b).

Among the top 25 most highly induced SEs, most were located in the vicinity of genes that are strongly expressed after macrophage activation (Fig. 4b, top panel). Many of these genes are known to be essential players in the macrophage inflammatory response, such as *Il1a*, *Il1b*, *Ccl3* and *Ccl5* [37, 38]. The most highly activated SE is near *Rsad2*, a gene encoding Viperin, an antiviral protein that is induced in primary human macrophages [39].

In contrast, many genes which maintain the naïve macrophage state are located near repressed SEs, and are expressed at lower levels following macrophage stimulation (Fig. 4b). For example, SLC29A3, SETDB1, and TNFAIP8L2 all serve to negatively regulate macrophages [40–43]. These findings provide further insight into the gene expression program that controls macrophage homeostasis.

Inhibitors of Brd4 slows tumor cell growth by blocking SE-mediated activation of cancer driver genes [3]. To determine whether the inflammatory response can be attenuated through inhibition of SEs in a similar manner, we looked at TLR4-stimulated macrophages that were exposed to the Brd4 inhibitor, iBET. SE-associated gene expression decreased in macrophages upon exposure to iBET (Fig. 4c, Additional file 9: Figure S4B). These findings provide further understanding as to why such a drug can confer protection against systemic inflammation [44].

SEs induced by TLR4 stimulation in macrophages initially exhibit weak P300 binding in resting macrophages, suggesting that many SEs originate at low density in unstimulated cells (Fig. 4b; examples of this at *Pim1*, *Gbp* gene cluster, *Cxcl10* and *Mir155* loci in Fig. 4d, Additional file 9: Figure S4C and S4D). eRNA expression coinciding with P300 peaks can also be seen at SEs in some of these examples (Fig. 4d, Additional file 9: Figure S4D). GBP proteins are critical in host defense and can activate the inflammasome [45–48].

We found that non-coding expression quantitative trait loci (eQTL) identified by Fairfax *et al*. [49], are often located within a monocyte SE (Fig. 4e, Additional file 9: Figure S4D-E), providing human genetic evidence for SE-mediated regulation. eQTLs are nearly twice as enriched in SEs, compared to CEs (54 % of SEs vs. 29 % of CEs) (Fig. 4f), although it remains a possibility that this enrichment is due to the fact that both SEs and eQTLs are enriched near promoters. As an example, we identify a SE that is associated with *SGK1*, which encodes a salt-sensing kinase recently implicated in Th17 development (Fig. 4e) [50]. The role of SGK1 in macrophages is not fully understood, although it has been implicated in disease [51]. Additional examples include *NOTCH1* and *LITAF*, which also perform important functions in macrophages (Additional file 9: Figures S4E and S4F).

## Discussion

By showing that P300 is a useful marker of SEs in diverse cell types, we have established a widely applicable approach for prioritizing functionally important genomic regulatory regions. We provide evidence that P300 SEs are generally similar to Med1 SEs in mESCs. The SEs we identified will be useful for understanding the regulation of genes important for defining cellular identity, which we demonstrate in our analysis of SEs and gene regulation in multiple cell state transitions. We report that during macrophage activation, the SE landscape is substantially remodeled, an observation that was not appreciated in previous studies [8, 9].

By showing that there are differences in the SE repertoire between resting and activated macrophage cells, we suggest that inhibiting these elements could be a useful strategy to curb inflammation in disease processes such as obesity-induced insulin resistance [52]. Specifically, genes regulated by SEs can be selectively repressed by Brd4 inhibitory drugs, such as iBET, with little effect on expression of other genes [3]. Genes that are induced by TLR4 signaling and associated with SEs in macrophages were strongly repressed by iBET. Since iBET and similar drugs are currently being clinically evaluated as anti-inflammatory and anti-cancer therapeutics, understanding their genetic targets in this way could lead to a better understanding of their mechanisms of action and possible side effects. In light of our findings, it would also be interesting to test P300-specific small molecule inhibitors [53].

SEs have been proposed to regulate genes important for cell identity. By examining P300 SEs in several immune cells and ESCs, we show that a large number of SEs are shared between closely related cells. However, genes important for cell identity, such as master regulator TFs, are often located near cell-specific SEs. These results are consistent with the notion that cell-specific SEs may play an important role in fate decision by regulating key genes, and could represent a novel approach for reprogramming cell fates. Interestingly, the BET inhibitor JQ1 has been demonstrated to inhibit Th17 cells in mouse and human [54]. Our work suggests that its mechanism of action may be to perturb SEs in Th17 cells. The findings in macrophages and Th17 cells suggest that BET inhibitors should be useful in curbing inflammation and autoimmunity. However, it has also been proposed to treat cancer, and it is important to monitor that those patients do not become immunodeficient.

Another acetyltransferase, CBP, a closely related paralog of P300, may mark a SE repertoire that is nearly identical to SEs found using P300. Often, P300 and CBP are considered synonymous and redundant [55, 56]. Due to the similarity between these proteins, the antibody used to detect P300 may also bind to CBP, and vice versa, a possibility that must be considered when interpreting our results. In humans, heterozygous inactivating mutation of CBP results in Rubinstein-Taybi Syndrome [57]. It would be interesting to determine whether pathogenesis of this disease is related to disruption of SEs. In addition, there is the P300/CBP-associated factor (PCAF) and its paralog, general control of amino acid synthesis nonrepressed protein 5 (GCN5), both lysine acetyltransferases. Their roles in relation to SEs are unknown.

It has been shown that transcription activity at enhancers is important for neighboring gene expression [58]. Several lncRNAs that were found to be needed for the maintenance of pluripotency in a recent screen [59], are identified here as associated with SEs. Similarly, the lncRNA NeST, which promotes *Ifng* expression and the CD8^+^ T cell response to infection by *Salmonella* [60], could be classified as a SE-associated lncRNA or eRNA. We found that SEs are widely transcribed relative to CEs, sometimes producing multiple distinct non-coding transcripts (although it does remain a possibility SEs are more likely than CEs to be active, which could also explain this observation). It is possible that many of these transcripts may be enhancer RNAs (eRNA), which may aid in enhancer regulation of nearby genes [61]. We propose that eRNAs emanating from SEs should be categorized as seRNAs. If these seRNAs contribute to SE function, they could represent an additional avenue for manipulating SE activity. Further, disease-associated genetic variation at SE sites could lead to disease by disrupting non-coding RNA and/or SE function.

## Conclusions

P300 ChIP-seq data can be leveraged to produce catalogs of SEs in diverse cell types and provide a useful resource to reveal key nodes in genetic regulatory networks that govern cell fate determination. Our findings illustrate the effectiveness of analyzing and integrating diverse datasets to make novel insights into biological processes, and our curated SEs will serve as an important resource to the scientific community.

## Methods

### ChIP-Seq analysis

All data sets used in this project are listed in Additional file 2: Tables S1 and Additional file 3: Table S2. ChIP-seq reads were mapped to either the mm9 build or hg19 build for mouse and human, respectively, using Bowtie (0.12.7) [62] and the following parameters: −m 2, −n 2, −k 1, −-best. Peaks for transcription factors and histone modifications were found using MACS (version 1.4.1) with the options –B, −n, −p 1e-9, −-keep-dup = auto [63]. Input control datasets and replicates were utilized whenever available (see Additional file 3: Table S2).

### Defining enhancers and super-enhancers

Enhancers and SEs were determined using a previously established approach [1]. Briefly, P300 ChIP-seq peaks with a *p*-value ≤ 1e-9 located within 12,500 base pairs from each other were stitched together to define enhancers as previously described for Med1 (with the only exception of mESC analysis in Fig. 1 that were performed using previously reported mouse enhancer sites [1]). Peaks located within 2,000 bp of a transcription start site were excluded. The ROSE algorithm [4] was used to calculate factor density within each enhancer, subtract input background, and rank enhancer regions via normalized read count. Enhancers were plotted with enhancer rank versus enhancer density, and all enhancer regions above the inflection point of the curve were defined as SEs.

### RNA-seq and GRO-seq analysis

RNA-seq reads were mapped to the mm9 genome using Tophat (version 2.011) [64]. Transcript abundances were estimated using HTSeq (version 0.6.1) [65]. Differential expression was calculated using DESeq (version 2.14) [66].

### Coding gene, LncRNA, miRNA, promoter, and eQTL overlap with enhancers

Genome coordinates of enhancer loci extended by 100 kb were compared to genome coordinates of protein coding, lncRNA and miRNA genes, as well as eQTLs. Any transcriptional elements that overlapped SEs by 1 bp or more were assigned to that SE. Gene coordinates were obtained from Ensembl release 65. lncRNA coordinates are from [26], miRNA coordinates are from miRBase 19. mESC promoters were defined by identifying H3K4me3 ChIP-seq peaks in mESCs and filtering for those which overlap any transcription start sites. All ternary plots were generated using the R package ggtern (version 1.0.3.2). The linkage disequilibrium (LD) region was calculated for each eQTL SNP using PLINK 1.9 [67] against the European individuals from the 1000 genomes reference panel [68].

### Genome browser screenshots

ChIP-seq and RNA-seq files were converted to the bigwig format, then uploaded as custom tracks to the UCSC genome browser [69]. Screenshots were downloaded and annotated in Adobe Illustrator. All scale bars for RNA-seq and ChIP-seq tracks contain 0 as the baseline (only the top scale bar is shown for each track to save space). Units are in tags per million, with the exception of the RNA-seq tracks in Fig. 2c, d, Fig. 3c, and e, which are in units of tags per total mapped reads. The plot and LD region for SNP rs12181791, rs7849014 and rs12446552 was calculated using the SNP Annotation and Proxy Search tool [70].

### Availability of supporting data

The datasets supporting the results of this article are included in Additional file 3: Table S2.

## Additional files

Additional file 1: Figure S1. A. All loci that were identified as SEs using both Med1 and P300 were compared by factor density. The correlation coefficient was determined to be 0.89. B. Average distance between enhancer regions and transcription start sites (TSS) for mESCs and other cells types. C. Average size of SEs. SEs were identified in mESCs using Med1, P300, and H3K27Ac ChIP-seq data. The average length in bp of each SE was calculated. ChIP-seq data are from [71, 72]. D. Example of a P300 SE in *Sox2* locus, a pluripotency gene, conserved between mouse and human ESCs. E. P300 SEs interacts physically with promoters in mESCs. Promoters were defined as H3K4me3 peaks that overlapped a transcription start site. Interactions between SEs and promoters were seen for 149 genes via Cohesin ChIA-PET, and 167 genes via RNA polymerase II ChIA-PET. 58 of these interactions were reproduced between both datasets. H3K4me3 data are from [1], Cohesin ChIA-PET is from [18], and RNA polymerase II ChIA-PET is from [20]. F. CBP marks a very similar SE repertoire as P300. SEs were determined from both P300 and CBP datasets in human T98 cells; 629/636 CBP SEs (98.9 %) were also identified from P300 data. Data are from [73]. (DOC 233 kb) Additional file 2: Table S1. Publicly available datasets used to curate SE catalogs.(XLSX 51 kb) Additional file 3: Table S2. A summary of datasets used in this study.(XLSX 14 kb) Additional file 4: Figure S2. A. SEs for Th1, Th2, and Th17 cells (from Fig. 3a) were grouped into modules based on module definitions in B. A Venn diagram was created based on these modules, depicting SEs that are either specific to a single type of cell or shared between two or three cell types. Data are from [26]. B. Cell specific modules. Genes, lncRNAs, miRNAs, and SEs were plotted based on relative expression (measured by RNA-seq, or ChIP-seq factor density for SEs calculated using the ROSE algorithm [1]) between three types of cells using the R package ggtern. Cell specific elements were defined as those with at least 60 % of expression or factor density in a single cell type (purple, red, and green areas). Elements shared between 2 or 3 types of cells were assigned to modules in a similar manner (see figure). C. Genes from both Th17 SE module and Th17 gene expression module have lower expression when missing the transcription factors *Stat3, Irf4, and C-maf*. CE-genes shown are unaffected. Data are from [25, 26]. D. The number constituent P300 peaks from Th17 SEs and TEs that overlap genomic regions containing all of the transcription factors STAT3, IRF4, BATF, c-MAF, and RORγt. Of 8,249 total genomic regions containing all 5 factors, 91 did not overlap either SEs or CEs. Data are from [25]. E. Comparison of Th17 specific genes that are associated with Th17 specific SEs or CEs reveals that SE associated genes are significantly more dependent on Stat3 for expression perturbation (*p*-value ≤ 0.019, one-tailed students *t*-test). Th17 specific genes located within 100 kb of a Th17 specific SE represent the ‘SE-associated Genes.’ Th17 specific genes within 100 kb of a CE represent the ‘CE-associated Genes.’ Data are from [25, 26]. (DOC 247 kb) Additional file 5: Table S3. Cell-specific SE-associated genes in T helper cells.(XLSX 74 kb) Additional file 6: Table S4. Cell-specific SE-associated lncRNAs in T helper cells.(XLSX 58 kb) Additional file 7: Table S5. Table S5: Cell-specific SE-associated miRNAs in T helper cells.(XLSX 35 kb) Additional file 8: Figure S3. A. A genome browser screenshot depicts a mouse SE which overlaps the *Ccr7* gene and lncRNAs (or eRNAs) that are enriched in Th17 cells. Expression of these lncRNAs in Th17 cells depend on Stat3 or Batf, and these two transcription factors bind extensively throughout this locus and co-localize with P300. This locus also contains sequence homology to two human Th17 cell-specific SEs [4], mapped by synteny. Data are from [25]. (DOC 100 kb) Additional file 9: Figure S4. A. All expressed genes in macrophages were assigned to either TLR4 induced SEs, pre-existing SEs, or no SE association. Fold change in expression following TLR4 stimulation was analyzed for these three groups of genes. B. iBET decreases the expression of SE associated genes in TLR4 stimulated macrophages. The genes located within 100 kb of the top 25 most highly induced SEs (following TLR4 stimulation) show a smaller increase in expression when treated with iBET (*p*-value = 0.001, Students *t* test). Gene expression fold changes were log2 transformed prior to plotting. C. A CE with low P300 density in resting macrophages becomes a SE in activated macrophages. There is also corresponding activation of expression of the genes *Gbp5*, *Gbp7*, *Gbp3*, and *Gbp2* that can be inhibited by iBET. D. (Top) An enhancer with low P300 density in resting macrophages becomes a SE in activated macrophages. There is a 292x fold increase in the expression of the nearby gene *Cxcl10* when the enhancer becomes an active SE. Data are from [36] and [34]. (Bottom) Two SEs near the *Mir155* are induced in activated macrophages. There is a 548x fold increase in the expression of *Mir155* primary transcript. E. (Top) Shown is a local association plot for a cis-eQTL that regulates the *NOTCH1* gene. The most significantly associated SNP from the linkage disequilibrium (LD) region is rs7849014. The majority of the LD region is also located within a CD14^+^ SE. eQTL data are from naïve CD14^+^ cells [49]. (Bottom) Expression of *NOTCH1* in CD14^+^ cells, along with H3K27ac density (data are from GSE18927). F. (Top) Shown is a local association plot for a cis-eQTL that regulates the *LITAF* gene. The most significantly associated SNP from the linkage disequilibrium (LD) region is rs12446552. The majority of the LD region is also located within a CD14^+^ SE that we identified. eQTL data are from naïve CD14^+^ cells [49]. (Bottom) Expression of *LITAF* in CD14^+^ cells, along with H3K27ac density (data from GSE18927). (PDF 105 kb)

## Abbreviations

BET: Bromodomain and extraterminal domain family
CE: Conventional enhancer
ChIA-PET: Chromatin interaction analysis by paired-end tag sequencing
ChIP-seq: Chromatin immunoprecipitation with DNA sequencing
eQTL: Expression quantitative trait loci
eRNA: Enhancer RNA
GRO-seq: Genomic run-on sequencing
lncRNA: Long non-coding RNA
mESC: Mouse embryonic stem cell
miRNA: MicroRNA
RNA-seq: RNA sequencing
SE: Super-enhancer
TF: Transcription factor
TSS: Transcription start site
