# Supplementary material for: High-density P300 enhancers control cell state transitions
Source: BMC Genomics. 2015 Nov 6;16:903. doi: 10.1186/s12864-015-1905-6 (PMC4636788; doi:10.1186/s12864-015-1905-6)

Supplemental Figure 1

A

Correlation of med1 and P300 binding density at SE sites in mESCs

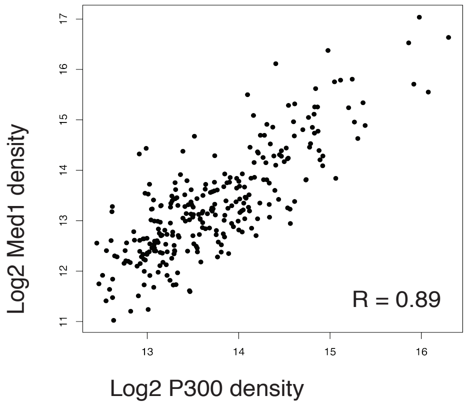

B

Cell type	ChIP-seq factor	Enhancer type	Number of Enhancers	Av. distance to nearest TSS (bp)	Standard deviation	P-value (CE vs. SE)	% enhancers overlapping TSS
mESC	Med1	CE	8563	74719	145509	3.8E-56	2.5%
mESC	Med1	SE	231	24568	34825		19.9%
Macrophage	P300	CE	4654	41716	70312	0.036	9.8%
Macrophage	P300	SE	123	31833	50076		35.8%
Macrophage LPS	P300	CE	4507	42333	71447	6.6E-07	9.5%
Macrophage LPS	P300	SE	144	23523	41646		39.6%
Th1	P300	CE	21063	43287	92401	1.2E-14	24.4%
Th1	P300	SE	1335	34529	81404		52.8%
Th2	P300	CE	21175	43287	92401	4.1E-06	24.7%
Th2	P300	SE	1223	33737	71529		51.5%
Th17	P300	CE	21401	43287	92401	0.00098	24.8%
Th17	P300	SE	997	29085	63721		53.8%

C

Average size of SEs identified with Med1, P300, and H3K27Ac

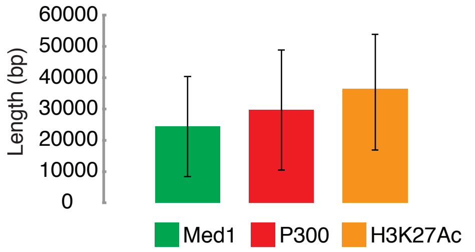

D

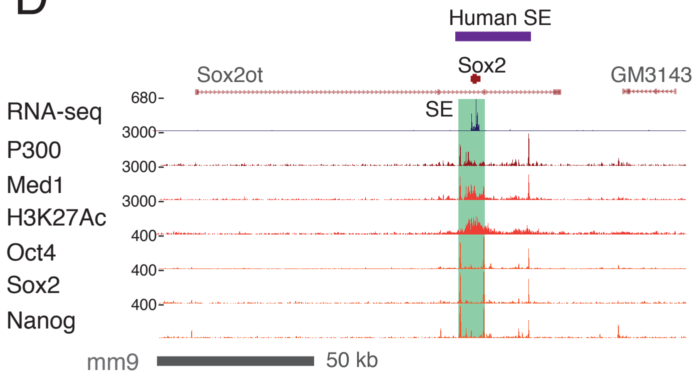

Supplement: Additional file 1: Figure S1. — A. All loci that were identified as SEs using both Med1 and P300 were compared by factor density. The correlation coefficient was determined to be 0.89. B. Average distance between enhancer regions and transcription start sites (TSS) for mESCs and other cells types. C. Average size of SEs. SEs were identified in mESCs using Med1, P300, and H3K27Ac ChIP-seq data. The average length in bp of each SE was calculated. ChIP-seq data are from [71, 72]. D. Example of a P300 SE in Sox2 locus, a pluripotency gene, conserved between mouse and human ESCs. E. P300 SEs interacts physically with promoters in mESCs. Promoters were defined as H3K4me3 peaks that overlapped a transcription start site. Interactions between SEs and promoters were seen for 149 genes via Cohesin ChIA-PET, and 167 genes via RNA polymerase II ChIA-PET. 58 of these interactions were reproduced between both datasets. H3K4me3 data are from [1], Cohesin ChIA-PET is from [18], and RNA polymerase II ChIA-PET is from [20]. F. CBP marks a very similar SE repertoire as P300. SEs were determined from both P300 and CBP datasets in human T98 cells; 629/636 CBP SEs (98.9 %) were also identified from P300 data. Data are from [73]. (DOC 233 kb) [file 12864_2015_1905_MOESM1_ESM.doc]
