# Supplementary figures and images for: High-density P300 enhancers control cell state transitions
Source: BMC Genomics. 2015 Nov 6;16:903. doi: 10.1186/s12864-015-1905-6 (PMC4636788; doi:10.1186/s12864-015-1905-6)

Figure S2

A

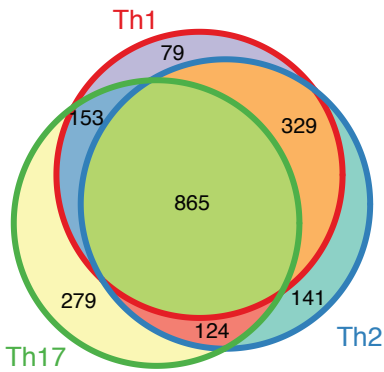

B

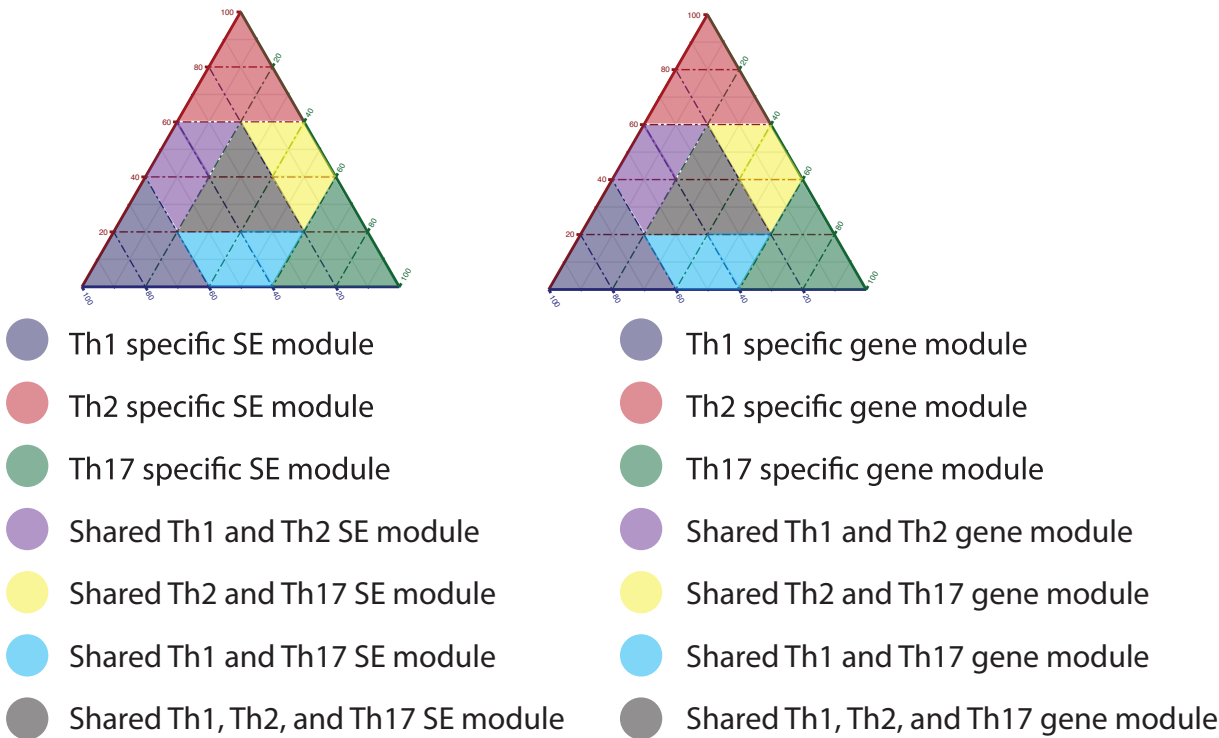

**Deletion of Key Transcription Factors Disrupts Expression of Th17 Genes**

C

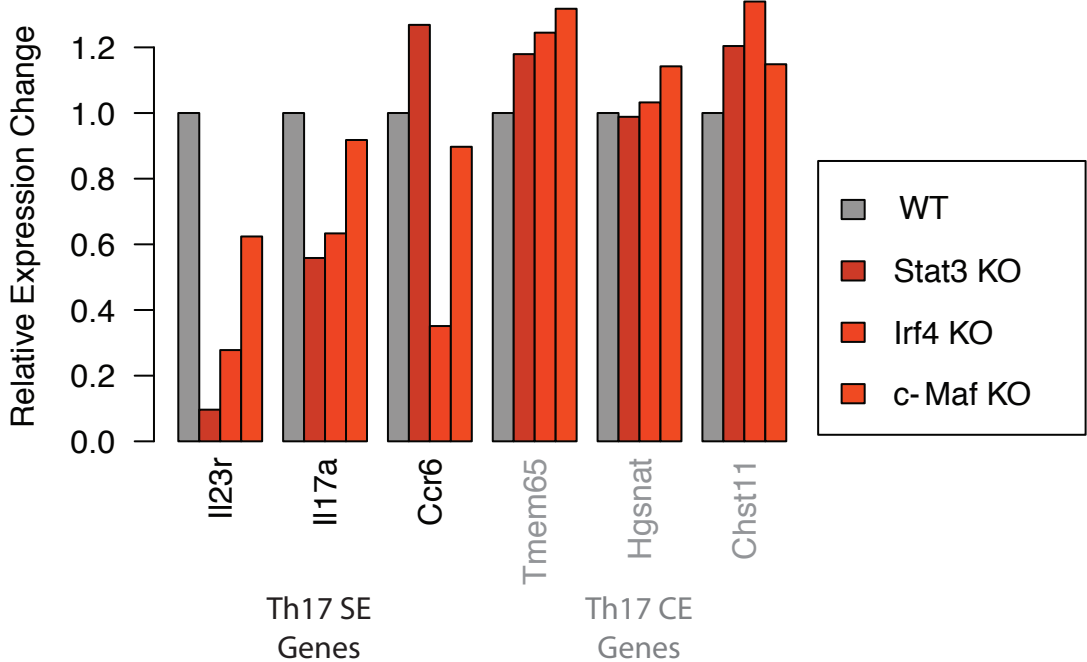

Supplement: Additional file 4: Figure S2. — A. SEs for Th1, Th2, and Th17 cells (from Fig. 3a) were grouped into modules based on module definitions in B. A Venn diagram was created based on these modules, depicting SEs that are either specific to a single type of cell or shared between two or three cell types. Data are from [26]. B. Cell specific modules. Genes, lncRNAs, miRNAs, and SEs were plotted based on relative expression (measured by RNA-seq, or ChIP-seq factor density for SEs calculated using the ROSE algorithm [1]) between three types of cells using the R package ggtern. Cell specific elements were defined as those with at least 60 % of expression or factor density in a single cell type (purple, red, and green areas). Elements shared between 2 or 3 types of cells were assigned to modules in a similar manner (see figure). C. Genes from both Th17 SE module and Th17 gene expression module have lower expression when missing the transcription factors Stat3, Irf4, and C-maf. CE-genes shown are unaffected. Data are from [25, 26]. D. The number constituent P300 peaks from Th17 SEs and TEs that overlap genomic regions containing all of the transcription factors STAT3, IRF4, BATF, c-MAF, and RORγt. Of 8,249 total genomic regions containing all 5 factors, 91 did not overlap either SEs or CEs. Data are from [25]. E. Comparison of Th17 specific genes that are associated with Th17 specific SEs or CEs reveals that SE associated genes are significantly more dependent on Stat3 for expression perturbation (p-value ≤ 0.019, one-tailed students t-test). Th17 specific genes located within 100 kb of a Th17 specific SE represent the ‘SE-associated Genes.’ Th17 specific genes within 100 kb of a CE represent the ‘CE-associated Genes.’ Data are from [25, 26]. (DOC 247 kb) [file 12864_2015_1905_MOESM4_ESM.doc]

E

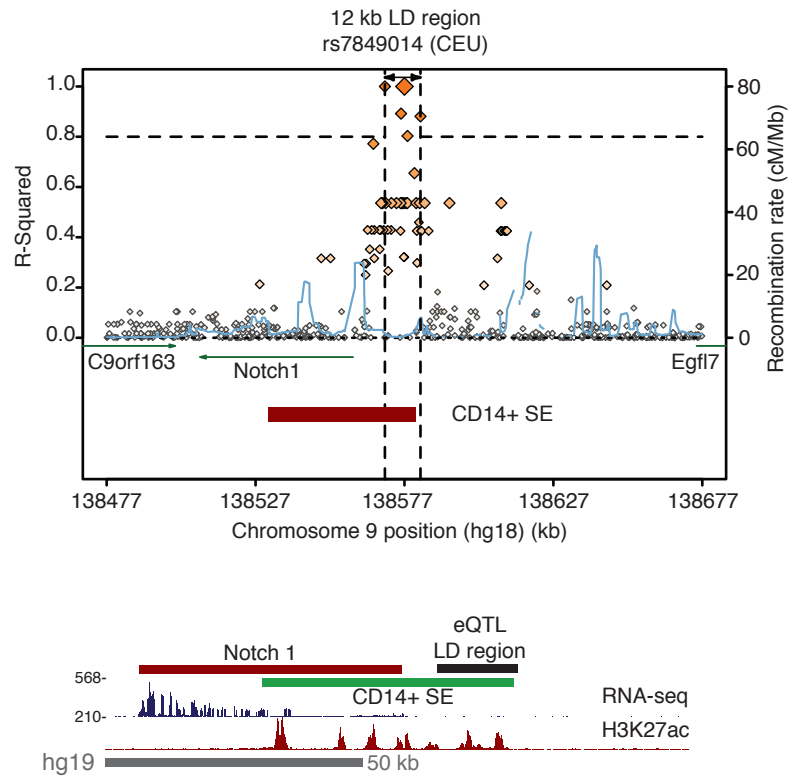

F

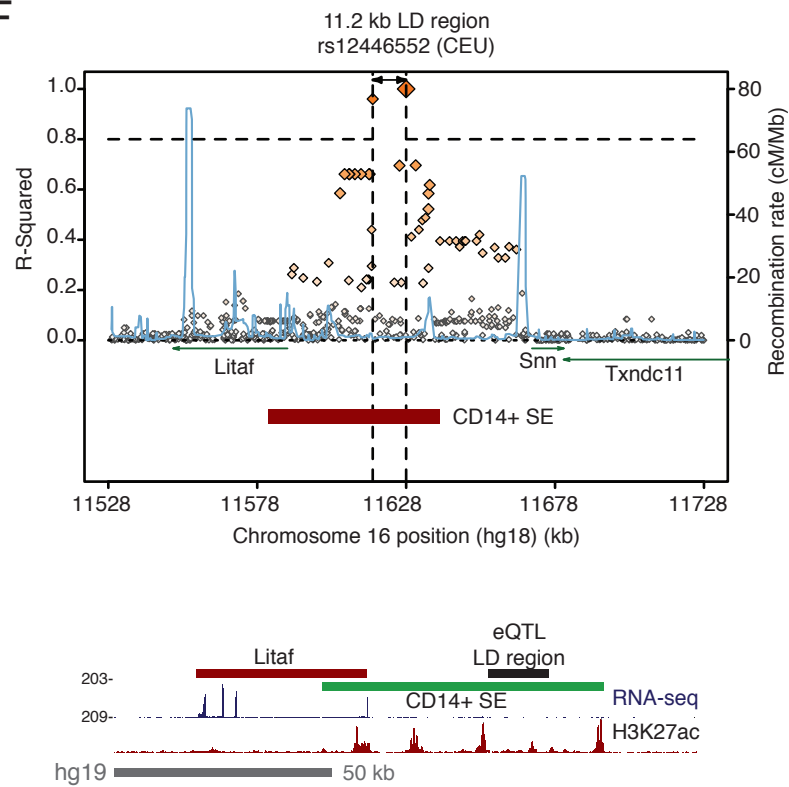

Supplement: Additional file 8: Figure S3. — A. A genome browser screenshot depicts a mouse SE which overlaps the Ccr7 gene and lncRNAs (or eRNAs) that are enriched in Th17 cells. Expression of these lncRNAs in Th17 cells depend on Stat3 or Batf, and these two transcription factors bind extensively throughout this locus and co-localize with P300. This locus also contains sequence homology to two human Th17 cell-specific SEs [4], mapped by synteny. Data are from [25]. (DOC 100 kb) [file 12864_2015_1905_MOESM8_ESM.doc]

**Figure S4**

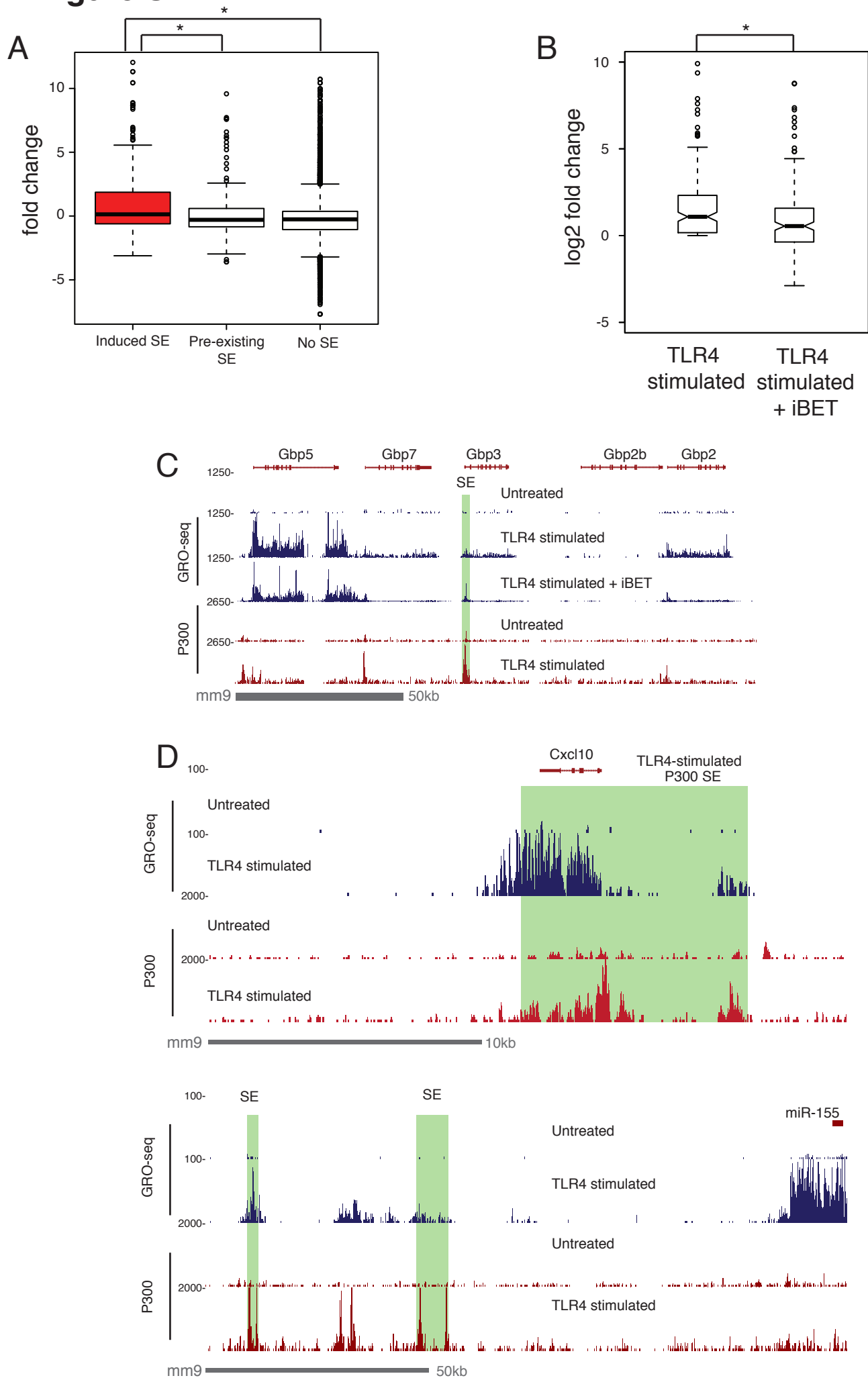

Supplement: Additional file 9: Figure S4. — A. All expressed genes in macrophages were assigned to either TLR4 induced SEs, pre-existing SEs, or no SE association. Fold change in expression following TLR4 stimulation was analyzed for these three groups of genes. B. iBET decreases the expression of SE associated genes in TLR4 stimulated macrophages. The genes located within 100 kb of the top 25 most highly induced SEs (following TLR4 stimulation) show a smaller increase in expression when treated with iBET (p-value = 0.001, Students t test). Gene expression fold changes were log2 transformed prior to plotting. C. A CE with low P300 density in resting macrophages becomes a SE in activated macrophages. There is also corresponding activation of expression of the genes Gbp5, Gbp7, Gbp3, and Gbp2 that can be inhibited by iBET. D. (Top) An enhancer with low P300 density in resting macrophages becomes a SE in activated macrophages. There is a 292x fold increase in the expression of the nearby gene Cxcl10 when the enhancer becomes an active SE. Data are from [36] and [34]. (Bottom) Two SEs near the Mir155 are induced in activated macrophages. There is a 548x fold increase in the expression of Mir155 primary transcript. E. (Top) Shown is a local association plot for a cis-eQTL that regulates the NOTCH1 gene. The most significantly associated SNP from the linkage disequilibrium (LD) region is rs7849014. The majority of the LD region is also located within a CD14+ SE. eQTL data are from naïve CD14+ cells [49]. (Bottom) Expression of NOTCH1 in CD14+ cells, along with H3K27ac density (data are from GSE18927). F. (Top) Shown is a local association plot for a cis-eQTL that regulates the LITAF gene. The most significantly associated SNP from the linkage disequilibrium (LD) region is rs12446552. The majority of the LD region is also located within a CD14+ SE that we identified. eQTL data are from naïve CD14+ cells [49]. (Bottom) Expression of LITAF in CD14+ cells, along with H3K27ac density (data from GSE18927). (PDF 105 kb) [file 12864_2015_1905_MOESM9_ESM.pdf]
